# Surfactant Protein B Deficiency Induced High Surface Tension: Relationship between Alveolar Micromechanics, Alveolar Fluid Properties and Alveolar Epithelial Cell Injury

**DOI:** 10.3390/ijms20174243

**Published:** 2019-08-30

**Authors:** Nina Rühl, Elena Lopez-Rodriguez, Karolin Albert, Bradford J Smith, Timothy E Weaver, Matthias Ochs, Lars Knudsen

**Affiliations:** 1 Institute of Functional and Applied Anatomy, Hannover Medical School, Hannover 30625, Germany; 2 Biomedical Research in Endstage and Obstructive Lung Diseases (BREATH), Member of the German Center for Lung Research (DLZ), Hannover 30625, Germany; 3 REBIRTH, Cluster of Excellence, Hannover 30625, Germany; 4 Institute of Vegetative Anatomy, Charite, Berlin 10117, Germany; 5 Department of Bioengineering, University of Colorado Denver, Denver, CO 80045, USA; 6 Division of Pulmonary Biology, University of Cincinnati College of Medicine, Cincinnati, OH 45221, USA

**Keywords:** surfactant protein B, atelectrauma, alveolar fluid, acinar micromechanics, acute lung injury

## Abstract

High surface tension at the alveolar air-liquid interface is a typical feature of acute and chronic lung injury. However, the manner in which high surface tension contributes to lung injury is not well understood. This study investigated the relationship between abnormal alveolar micromechanics, alveolar epithelial injury, intra-alveolar fluid properties and remodeling in the conditional surfactant protein B (SP-B) knockout mouse model. Measurements of pulmonary mechanics, broncho-alveolar lavage fluid (BAL), and design-based stereology were performed as a function of time of SP-B deficiency. After one day of SP-B deficiency the volume of alveolar fluid V(alvfluid,par) as well as BAL protein and albumin levels were normal while the surface area of injured alveolar epithelium S(AEinjure,sep) was significantly increased. Alveoli and alveolar surface area could be recruited by increasing the air inflation pressure. Quasi-static pressure-volume loops were characterized by an increased hysteresis while the inspiratory capacity was reduced. After 3 days, an increase in V(alvfluid,par) as well as BAL protein and albumin levels were linked with a failure of both alveolar recruitment and airway pressure-dependent redistribution of alveolar fluid. Over time, V(alvfluid,par) increased exponentially with S(AEinjure,sep). In conclusion, high surface tension induces alveolar epithelial injury prior to edema formation. After passing a threshold, epithelial injury results in vascular leakage and exponential accumulation of alveolar fluid critically hampering alveolar recruitability.

## 1. Introduction

The intra-alveolar surfactant is a mixture of 90% lipids (mainly phospholipids) and 10% proteins including surfactant proteins which are produced, stored and secreted by alveolar epithelial type 2 (AE2) cells [1]. Among the protein component the hydrophobic surfactant proteins B (SP-B) and C (SP-C) are both of high relevance for the surface tension lowering properties of alveolar surfactant at the air-liquid interface within the alveolar space. Underneath this interface, a very thin layer of liquid, also referred to as the hypophase, covers the alveolar epithelium [2,3]. This reduction in surface tension is critical to prevent end-expiratory collapse of distal airspaces and to reduce the work of breathing. Comparing quasi-static pressure-volume (PV) loops of air and liquid-filled healthy lungs it becomes obvious that the hysteresis, e.g., the area within the PV-loop, is mainly a feature of the air-liquid interface. The corresponding surface phenomena such as surface tension, moreover, result in larger pressures needed for lung inflation and a loss of energy during the PV-loop as characterized by the hysteresis [4]. The surface active molecules, such as phospholipids, form a dynamic layer at the air-liquid interface. This layer is mechanically challenged by the cyclic intra-tidal changes in the geometry of alveoli and interalveolar septa which expand during inspiration and form pleats at the end of expiration [5]. Accordingly, the surfactant layer at the air-liquid interface is compressed at end-expiration and reduces the surface tension close to 0 mN/m. During inspiration the surface active film of phospholipids is expanded and the surface tension increases at larger lung volumes [6,7]. SP-B, due to its properties to generate phospholipid membrane-membrane contacts, has been suggested to be involved in the formation and stabilization of surface active films so that surface tension lowering properties are critically dependent on the biophysical properties of SP-B [2,8].

The acute respiratory distress syndrome (ARDS) is characterized by a severe failure of the lungs’ central function in gas exchange resulting from an injury of the blood-gas barrier. Accordingly, inflammation, alveolar flooding with surfactant inactivation and alveolar collapse are typical features of ARDS [9,10]. The high surface tension itself has effects on the acinar microarchitecture including its dynamic changes during respiration, also known as alveolar micromechanics. Based on the Wilson-Bachofen model, surface tension at the air-liquid interface results in forces which would induce a piling up of interalveolar septal walls and therefore a reduction in surface area [11]. These surface tension-related forces are counter-balanced by the axial system of elastic fibers which usually surround the alveolar entrance. High surface tension induced by lavage of the lung with tween has been shown to result in a diversity of abnormalities in alveolar micromechanics such as intratidal alveolar recruitment/derecruitment [12] but also asynchronous alveolar dynamics such as inverse alveolar ventilation, alveolar stunning or alveolar pendelluft [13].

Clinical studies demonstrated that a reduction in the SP-B level in the alveolar space represents an early event during the time course and can even precede the development of the complete clinical picture of ARDS [14,15]. In established ARDS, moreover, the level of SP-B in broncho-alveolar lavage fluid (BAL) correlated convincingly with the impairment in surfactant function as characterized by the minimum surface tension [16]. However, in the context of clinical ARDS, the relevance of this early SP-B reduction has not been investigated in detail although it has been suggested that surfactant inactivation plays a central role in the development of ARDS [17]. It is well known that high surface tension leads to edema formation [18] and expiratory alveolar derecruitment [19]. In the conditional SP-B knockout mouse model high surface tension results in pulmonary inflammation, respiratory failure and death [20,21]. However, the mechanisms leading to respiratory failure in SP-B deficiency-induced high surface tension are not entirely understood. Several models of pulmonary micromechanics offer realistic scenarios by which SP-B deficiency induced high surface tension can result in progressive lung injury [22,23,24,25]. The failure of surfactant to reduce surface tension during expiration results in alveolar instability with derecruitment of alveolar surface area or even complete alveoli which can be recruited if transpulmonary pressure gradients increase again during inspiration [26]. This repetitive intratidal alveolar recruitment/derecruitment has been observed in lavage models of acute lung injury during mechanical ventilation [12]. There is evidence that repetitive recruitment/derecruitment of distal airspaces can be harmful to the alveolar epithelium thereby contributing to ventilation-induced lung injury (VILI) via a mechanism known as atelectrauma [27]. Computational modelling combined with experimental validation in cell culture model systems provided evidence that the opening of a fluid-occluded distal airspace can, in the presence of high surface tension, be associated with potential harmful pressure gradients acting on the epithelial lining [22]. Accordingly, restoring surfactant function and reducing surface tension protected the epithelial lining in models of fluid occluded distal airspace recruitment [22,28]. It has also been shown that the properties of the air-liquid interface are critical for the function of AE2 cells because the surface tension forces exert deforming mechanical stresses (e.g., shear stress and tensile strain) on the AE2 cells [23,24,29]. As a result AE2 cells change gene expression profiles in a way that resembles VILI, cyclic alveolar stretch, and pulmonary fibrosis [23].

Transferring these micromechanical models of atelectrauma [22] and interfacial stresses [23] into the context of SP-B deficiency-induced high surface tension it can be hypothesized that repetitive opening of distal airspaces containing fluid (= hypophase) during breathing might be an initial trigger for injury of the alveolar epithelium. These injuries may represent the initial injurious event that occurs prior to vascular leak and alveolar edema accumulation. On the other hand, high surface tension has also been suggested to result in alveolar edema [18] so that the initial consequence of SP-B deficiency could be alveolar fluid accumulation and heterogeneous alveolar ventilation. Discrete alveolar flooding has been shown to be sufficient to induce epithelial injury characterized by vascular leakage due to overdistension of neighboring alveolar airspaces even during ventilation with quite low tidal volumes [30]. Therefore, injury of the alveolar epithelium and progressive respiratory failure might be the consequence, and not the cause, of edema formation.

Based on these considerations the goal of the present study was to understand the relationship between impaired acinar micromechanics, injury of the alveolar epithelium and the alveolar fluid properties in SP-B deficiency induced high surface tension. For this purpose, the time course of these different pathologies was investigated after depletion of SP-B in a mouse model expressing SP-B under control of a doxycycline dependent promotor [21]. Doxycycline-containing food was withdrawn and animals were investigated 1 (group: Dox off d1) and 3 (group: Dox off d3) days thereafter using lung mechanical and BAL parameters and quantitative morphology based on design-based stereology. The latter included a protocol of vascular perfusion fixation of the lungs at airway opening pressures (Pao) on expiration of 2 and 10 cmH_2_O in order to investigate the recruitability of distal airspaces. Alveolar microarchitecture was described by stereological parameters such as the total volume of alveolar airspaces, the number of open alveoli, the total surface area of alveoli and the mean thickness of interalveolar septal walls. For the assessment of lung injury, the surface area of the epithelial basal lamina covered by injured cells (based on ultrastructural criteria) was determined. Finally, the intra-alveolar fluid was characterized by its absolute volume per lung, the mean thickness and the surface area of the alveolar epithelial cells covered by fluid. As additional parameters lung mechanical data and protein levels in BAL were determined. The data of lungs from mice after withdrawal of doxycycline containing food (Dox off) were compared to those of lungs from mice having been fed with the doxycycline containing food (Dox on).

## 2. Results

### 2.1. Lung Mechanics

**Figure 1:**
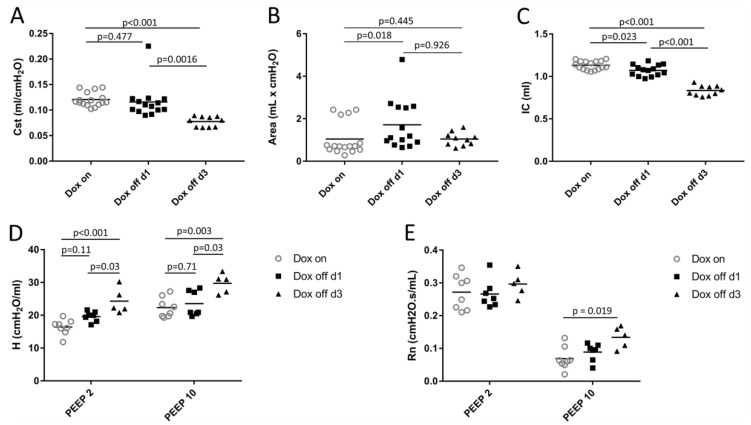
Lung mechanical properties. Quasi-static compliance (**A**), and hysteresis of quasi-static PV loops (**B**), inspiratory capacity (**C**), tissue elastance (**D**), and Newtonian resistance (**E**). PEEP: positive end-expiratory pressure.

Data describing lung mechanical function are detailed in Figure 1. The quasi-static compliance (Cst) is the slope of the deflation limb of a quasi-static PV-loop at an airway opening pressure of 5 cmH_2_O and was significantly reduced in Dox off d3 compared to both the Dox on and the Dox off d1 groups (Figure 1A). However, a difference regarding this parameter was not observed between Dox on and Dox off d1. Quasi-static PV-loops were further investigated and the hysteresis (= area within the PV loop) was determined (Figure 1B). While hysteresis in Dox off d1 was significantly increased compared to Dox on this was not the case considering Dox off d3. Inspiratory capacity (IC) is defined as the volume of displaced air into the lung during a ramp inflation from 3 to 30 cmH_2_O over a period of 6 s. Unlike Cst, IC showed a significant decrease in Dox off d1 compared to Dox on (Figure 1C). Dox off d3 was characterized by an even more dramatic reduction in IC compared to Dox on and Dox off d1 (Figure 1C).

As a forth parameter, the tissue elastance H, was determined using the forced oscillation technique (FOT) fit to the constant phase model [31] during ventilation at positive end-expiratory pressures (PEEP) of 2 and 10 cmH_2_O. The tissue elastance H reflects the lung mechanics at the different PEEP levels because the onset pressure of the FOT corresponded to the PEEP level and since the FOT volume variations were quite small (3ml/kg bodyweight). As such, tissue elastance H takes the degree of end-expiratory airspace collapse into consideration [19,26,32,33]. At both PEEP levels (2 and 10 cmH_2_O) there was no significant difference between Dox on and Dox off d1 Figure 1D). Dox off d3, however, demonstrated a significant increase in H compared to both other groups at both PEEP levels (Figure 1D). The Newtonian resistance (Rn) is also determined from the constant phase model fit to the FOT measurements. This parameter reflects pathologies of the conducting airways and shows a clear dependence on the PEEP at which it was determined (Figure 1E). The decrease of Rn with increasing PEEP can be explained by interdependence of conducting airways and surrounding lung parenchyma [34]. Outward tethering forces of the elastic fiber system which connects the conducting airways and the pleura increase with lung volume (and PEEP) so that the resistance of the conducting airways is reduced. At PEEP = 2 cmH_2_O there were no significant differences in Rn between study groups. However, increasing the PEEP from 2 to 10 cmH_2_O provided a smaller reduction in Rn for Dox off d3 so that a significant difference became apparent at PEEP 10 cmH_2_O compared to Dox on (Figure 1E).

### 2.2. Progressive Disturbances of Acinar Micromechanics in Dox off Groups

**Figure 2:**
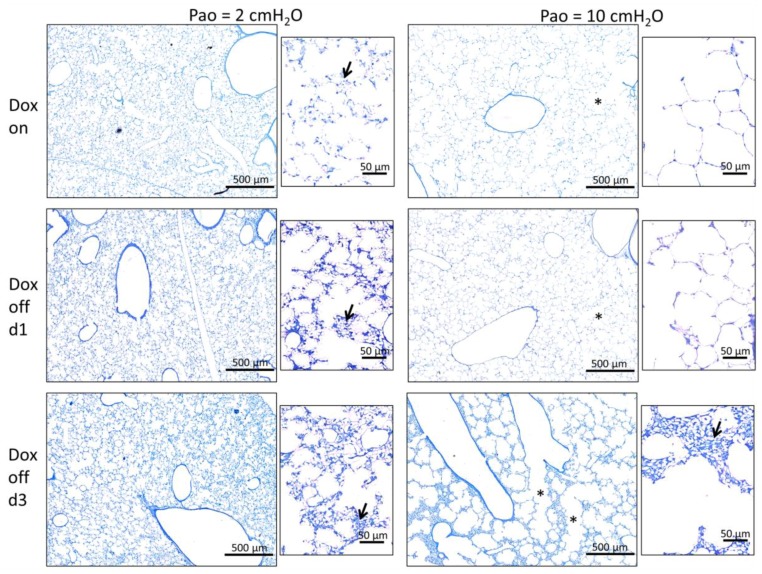
Microatelectases. Representative light microscopic images of Dox on, Dox off d1 and Dox off d3 of lung tissue fixed at end-expiratory airway opening pressure (Pao) of 2 and 10 cmH_2_O. The arrows indicate piled septal walls due to microatelectases which can be observed at Pao = 2 cmH_2_O in all study groups, while at Pao = 10 cmH_2_O microatelectases are present in Dox off d3 but not Dox on or Dox off d1. In addition, the alveolar ducts appear enlarged in some regions (asterisk) in Dox off d3 at Pao = 10 cmH_2_O. Arrow: microatelectasis; asterisk: alveolar duct.

Figure 2 illustrates representative light microscopic images of the study groups fixed by vascular perfusion at different end-expiratory airway opening pressures (Pao 2 cmH_2_O and 10 cmH_2_O). At the time point of fixation the lungs were air-filled so that the air-liquid interface was present and the effects of interfacial surface tension on lung structure could be investigated [26,35]. In general, the blood vessels including the capillary network within the interalveolar septa were free of blood cells and open so that it can be concluded that the perfusion fixation was successful. At lower magnification there were no apparent differences between Dox on (Figure 2) and Dox off d1. At higher magnification both study groups demonstrated signs of collapsed alveoli (microatelectases) and formation of pleats of interalveolar septal walls at Pao = 2 cmH_2_O which disappeared at Pao = 10 cmH_2_O. On contrary, microatelectases were present at both Pao = 2 cmH_2_O and Pao = 10 cmH_2_O in Dox off d3. Moreover, the alveolar ducts were widened while the interalveolar septa appeared to be piled up at Pao = 10 cmH_2_O (Figure 2).

**Figure 3:**
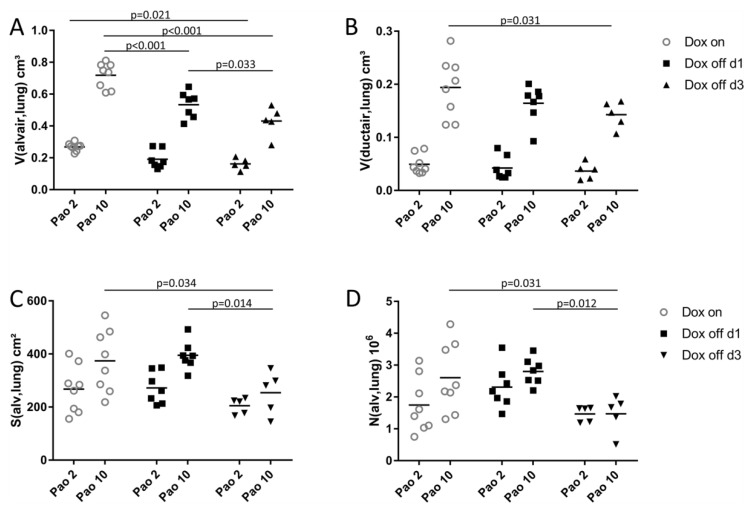
Acinar microarchitecture. The total volumes per lung of alveolar (**A**) V(alvair,lung) and ductal airspaces (**B**) V(ductair,lung) are given. In (**C**) the surface area of alveoli per lung S(alv,lung) and in (**D**) the total number of alveoli per lung N(alv,lung) are illustrated.

The stereological data regarding the acinar microarchitecture are summarized in Table 1. Highly significant effects of the duration of the withdrawal of doxycycline containing food as well as Pao on the total lung volume V(lung) could be identified. Having a closer look at the components of the lung parenchyma elucidated that the total volume of alveolar airspaces V(alvair,lung), (Figure 3A) was affected by the factor Dox off and Pao while the total volume of ductal airspaces V(ductair,lung) (Figure 3B) was effected by Pao only. The total volume of septal wall tissue V(sep,lung) was independent of both Dox off and Pao. At Pao of 2 cmH_2_O the differences in V(alvair,lung) between Dox on and the Dox off groups were small and there was only a significance between Dox on and Dox off d3 (Figure 3A). At larger Pao, however, the differences in V(alvair,lung) become more prominent with a highly significant difference between Dox on and Dox off d1 as well as between Dox off d1 and Dox off d3. The total surface area of alveoli S(alv,lung) and the total number of alveoli N(alv,lung) followed similar trends and did not show significant differences between Dox on, Dox off d1 and Dox off d3 at low Pao. At higher Pao, however, the Dox off d3 group was characterized by reduced S(alv,lung) (Figure 3C) and N(alv,lung) (Figure 3D) compared to both Dox on and Dox off d1. Based on the stereological findings illustrated in Figure 3 it can be summarized that one day after induction of SP-B deficiency the lungs have reduced alveolar airspaces at 10 cmH_2_O, a finding which is in line with the inspiratory capacity (Figure 1C). This indicates that the alveolar airspaces during inspiration are stiffer in Dox off d1. Three days after induction of SP-B deficiency there is progressive impairment of parenchymal recruitment with increasing Pao as reflected in the reduced alveolar airspace volume, alveolar surface area, and total number of open alveoli. Hence, the Dox off d3 group fails to recruit surface area and collapsed alveoli as Pao is increased.

### 2.3. Composition of Inter-alveolar Septa and Intra-alveolar Fluid Properties

The composition of the interalveolar septal walls was analyzed in detail (Table 2) at the electron microscopic level. The factor “group assignment” did not influence total volumes of cellular and extracellular components within the septal walls. This was also the case regarding the total volume of capillary lumen within the interalveolar septa. Effects of Pao were noted in the absolute volumes of extracellular matrix V(ECM,sep) and capillary lumen V(caplumen,sep) as well as the total surface area of the endothelial basal lamina S(endoBL,sep). Higher Pao resulted in a highly significant decrease in V(caplumen,sep) (Table 2) which can be explained by the compression of the septal walls due to higher pressure gradients between the alveolar airspaces and the capillary lumen. The significant increase in S(endoBL,sep) due to higher Pao can be explained by a higher degree of stretch at Pao = 10 cmH_2_O compared to Pao = 2 cmH_2_O. The behavior of V(ECM,sep), however, which shows larger values at Pao = 10 cmH_2_O compared to Pao = 2 cmH_2_O, is difficult to understand but might result from alterations in the distribution of water within the interstitial space.

**Figure 4:**
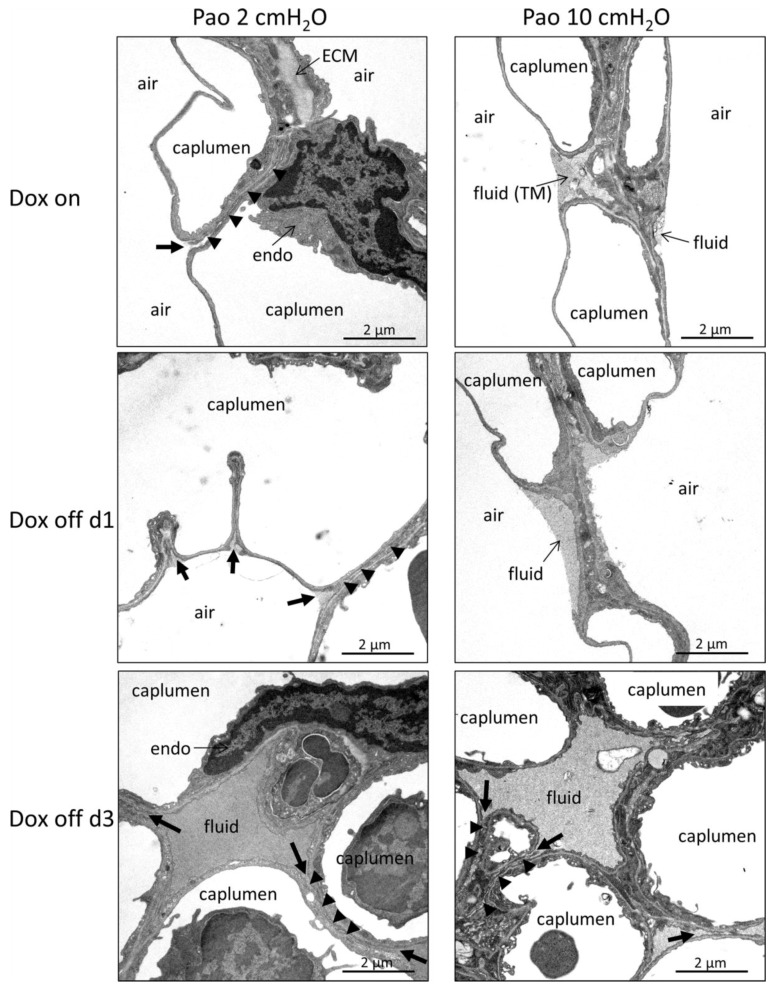
Alveolar fluid and septal wall folding. Electron microscopic images of lungs of Dox on, Dox off d1 and Dox off d3 fixed at positive end-expiratory airway opening pressure (Pao) of 2 and 10 cmH_2_O. Septal wall folding can be observed in all study groups at Pao = 2 cmH_2_O. The “entrances” to the folds are illustrated by arrows. The opposing septal walls are separated by very thin layers of alveolar fluid (arrowhead). At higher Pao, septal wall folds become less frequent and alveolar fluid can be seen in the corners of the alveoli in Dox on and Dox off d1. In Dox off d3, the amount of alveolar fluid seems to be increased and the occurrence of septal wall folds does not seem to be less frequently at higher Pao. Abbreviations: caplumen: lumen of septal wall capillaries; endo: endothelial cell; ECM: extracellular matrix; TM: tubular myelin, a lattice of membranes corresponding to an active intra-alveolar surfactant component within the hypophase. Black bold arrow: entrance to fold; arrowhead: liquid filled space between opposing epithelial cells.

**Figure 5:**
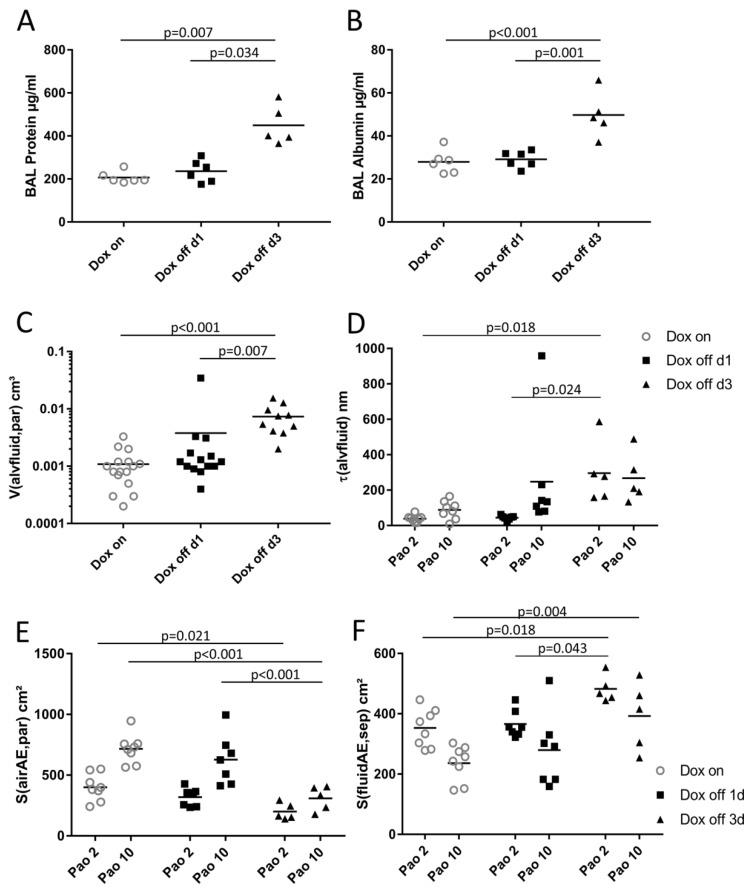
Properties of alveolar fluid. BAL was assessed regarding protein (**A**) and albumin (**B**) concentration. In addition, alveolar fluid was characterized by stereological parameters as follows: Total volume of alveolar fluid per lung (**C**) V(alvfluid,par), arithmetic mean thickness of alveolar fluid (**D**) τ(alvfluid), the surface area of alveolar epithelium covered by air (**E**) S(airAE,par) or covered by alveolar fluid (**F**) S(fluidAE,sep).

Electron microscopy was further used to study intra-alveolar fluid morphology as a function of the duration of withdrawal of doxycycline containing food and Pao. Figure 4 illustrates representative electron microscopic images of Dox on, Dox off d1 and Dox off d3 from lung tissue fixed at Pao 2 or 10 cmH_2_O. At Pao = 2 cmH_2_0 the formation of pleats of the septal walls was a typical finding in all study groups. A very thin layer of alveolar fluid could be identified between opposing alveolar epithelial cells. In some areas of Dox off d3 these layers of alveolar fluid were much darker and thicker compared to Dox on and Dox off d1. Increasing the Pao to 10 cmH_2_O was linked with a dramatic decrease in the frequency of septal wall pleats in Dox on and Dox off d1. The alveolar fluid was concentrated in the corners of alveoli and could be clearly identified at electron microscopic level. In group Dox off d3, however, pleats of septal walls in concert with quite thick layers of dense alveolar fluid interposing the space between the alveolar epithelial cells remained a quite common finding. Protein and albumin concentration in BAL were determined to assess alveolo-capillary barrier disruption [9]. While there were no significant differences in protein content between Dox on and Dox off d1, Dox off d3 demonstrated significantly increased levels compared to the other 2 groups (Figure 5A). These findings were in line with the BAL albumin level which was also significantly higher in Dox off d3 compared to Dox on and Dox off d1 (Figure 5B). Using design-based stereological methods at electron microscopic level, the total volume of alveolar fluid V(alvfluid,par), the arithmetic mean thickness of alveolar fluid τ(alvfluid), and the surface area of alveolar epithelium covered by air S(airAE,par) or by fluid S(fluidAE,par) were quantified. Data regarding the alveolar fluid are provided in Figure 5 and illustrated in Table 3.

The duration of withdrawal of doxycycline containing food but not the Pao demonstrated significant effects on V(alvfluid,par) and therefore Figure 5C illustrates group effects only. Compared to Dox on and Dox off d1 there was a significant increase in V(alvfluid,par) in Dox off d3 (Figure 5C) which was accompanied by a significant increase in the thickness of the fluid layer τ(alvfluid) (Figure 5D). Having a closer look at the Pao effects in the different groups it appeared that there is an increase in τ(alvfluid) with Pao in Dox on and Dox off d1 but not in Dox off d3 (Figure 5D). This observation might be attributed to a redistribution of fluid in Dox on and Dox off d1 during recruitment. Derecruited septal walls or even derecruited alveoli have a thin layer of alveolar fluid between opposing alveolar epithelial cells which, after the recruitment process, is concentrated in the alveolar corners resulting in a thickening of the fluid layer while its total volume remains roughly stable (Figure 4).

In line with these observations were S(airAE,par) and S(fluidAE,par). While S(airAE,par) could be increased by raising Pao from 2 to 10 cmH_2_O in Dox on and Dox off d1 this was not the case in Dox off d3 (Figure 5E). The parameter S(fluidAE,par) behaved exactly the other way round (Figure 5F) and decreased with increasing Pao in Dox on and Dox off d1. Accordingly, there were only small differences in S(airAE,par) at Pao 2 cmH_2_O between the Dox on and Dox off d3. By contrast, at Pao 10 cmH_2_O the surface area of air covered alveolar epithelium was significantly lower in Dox off d3 compared to both Dox on and Dox off d1 (Figure 5E). A similar trend was observed with S(fluidAE,par). By increasing Pao from 2 to 10 cmH_2_O, the differences between Dox off d3 and the other groups became larger (Figure 5F). In summary, the data obtained at electron microscopic level show a failure in the Dox off d3 group to recruit air covered alveolar epithelium with increasing Pao. This finding is in line with the light microscopic data. Moreover, the electron microscopical data demonstrate an increase in the volume and thickness of alveolar fluid in Dox off d3. Since the thickness and the surface area of alveolar epithelium covered by fluid show hardly any Pao effects in Dox off d3 it can be concluded that high surface tension or viscosity is linked with a failure to redistribute with changing Pao. These structural observations correlate with the increased BAL levels of protein and albumin in Dox off d3.

### 2.4. Ultrastructural Evaluation of Alveolar Epithelial Injury

**Figure 6:**
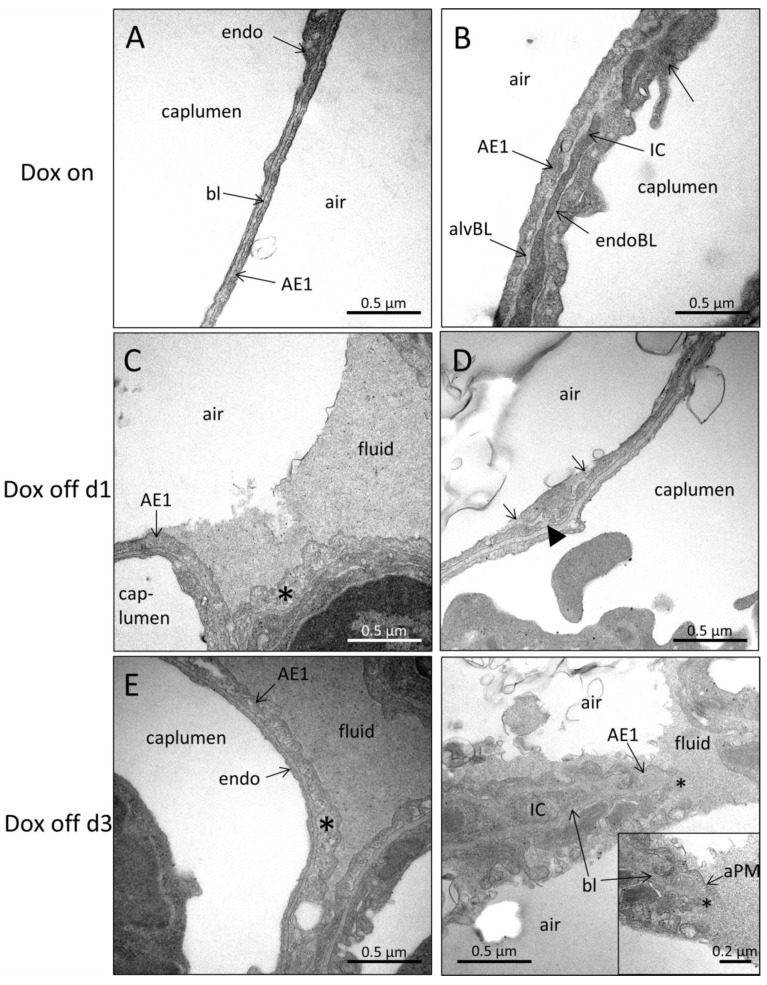
Injury of alveolar epithelial cells. Normal alveolar epithelial cells can be found in Dox on (**A**) and (**B**). In Dox off d1, swelling and clearance of cytoplasmic ground substance (asterisk) and ruptures in alveolar epithelial cell lining (arrowhead) could be found (**C**) and (**D**). In Dox off d3, similar observations as in Dox off d1 were present (**E**) and (**F**). Abbreviations: AE1: alveolar epithelial type 1 cell; endo: endothelial cell; caplumen: lumen of septal wall capillary; bl: basal lamina (junction of alvBL and endoBL); alvBL: alveolar epithelial basal lamina; endoBL: endothelial basal lamina; IC: interstitial cell; aPM: apical plasma membrane; air: alveolar airspace; fluid: alveolar fluid. Arrowhead: rupture of alveolar epithelial cell; asterisk: swollen AE1 cell.

The increase in BAL albumin in Dox off d3 can be attributed to disruption of the blood-gas barrier consisting of the alveolar epithelial cells, the basal lamina (= interstitium) and the endothelial cells. Previous studies have shown that the surfactant dysfunction in this animal model is present at 1 day after doxycycline withdrawal and remains consistent through day four [21]. High surface tension, moreover, has been shown to result in interfacial stress linked to injury of alveolar epithelial cells [22,36,37]. In order to understand whether Dox off d1 group shows in absence of elevated BAL protein alveolar epithelial injury the further ultrastructural investigation focused on alveolar epithelial cells. There were no obvious alterations in the ultrastructure of AE2 cells including the morphology of lamellar bodies. However, subtle abnormalities were observed regarding the AE1 cells. In Dox off d1 AE1 cells occasionally showed swelling and clearing of the cytoplasmic ground substance (Figure 6C). In healthy controls the AE1 cells were usually not swollen and the cytoplasmic ground substance was characterized by the same density as the endothelial cells or interstitial cells (Figure 6A,B). Furthermore, there were signs of rupture of the apical plasma membrane of AE1 cells in Dox off d1 (Figure 6D). Ruptures of the apical plasma membrane, swelling, and clearing of cytoplasmic ground substance were also observed in Dox off d3 AE1 cells (Figure 6E,F).

**Figure 7:**
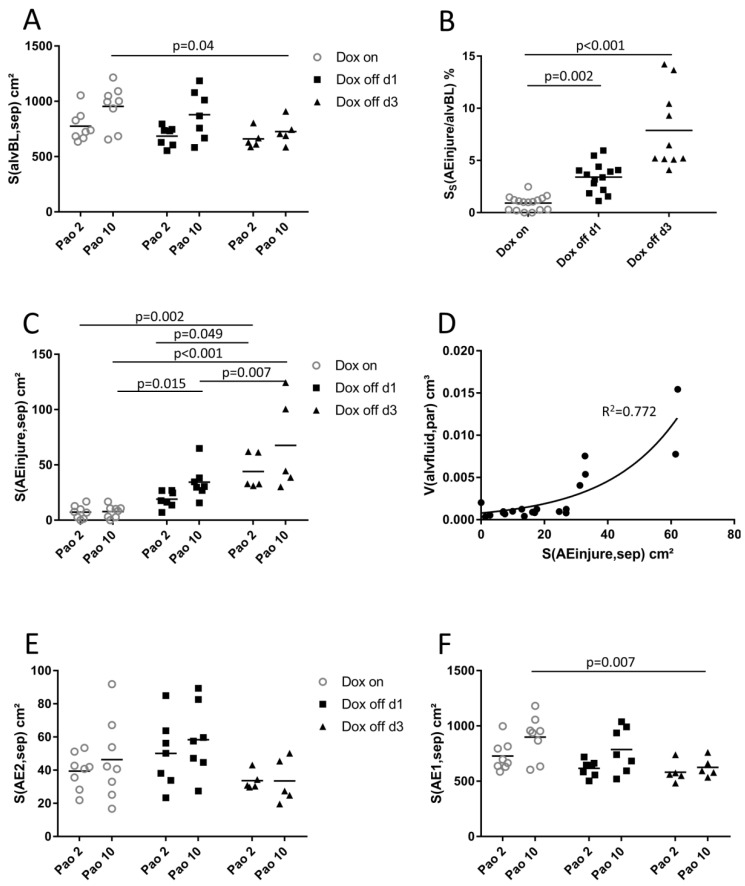
Stereological parameters of alveolar epithelial cell injury. Data were related to the total surface area of the alveolar epithelial basal lamina within septal walls (**A**) S(alvBL,sep). In (**B**), the surface fraction of the basal lamina covered by injured alveolar epithelial cells S_S_(AEinjure/alvBL) shows an increase in Dox off d1 and Dox off d3. Multiplication of the surface fraction and S(alvBL,sep) resulted in absolute surface areas within the septal walls of injured alveolar epithelial cells (**C**), normal appearing alveolar epithelial type 2 (**E**) and alveolar epithelial type 1 (**F**) cells. (**D**) demonstrates the relationship between the total surface area of injured alveolar epithelial cells S(AEinjure,sep) and the total volume of alveolar fluid V(alvfluid,par) which can be described by an exponential growth function.

Hence, a quantification of alveolar epithelial injury was performed (Table 4 and Figure 7). The total surface area of the alveolar epithelial basal lamina S(alvBL,sep) was found to increase with Pao (Figure 7A). This observation can be explained by Pao-induced stretching of alveolar septal walls. The surface fraction of the basal lamina covered either by injured epithelial cells S_S_(AEinjure/alvBL), healthy appearing AE1 cells S_S_(AE1/alvBL), or healthy appearing AE2 cells S_S_(AE2/alvBL) was determined in order to describe injury severity. Surface fractions were independent of Pao effects and influenced by group assignment. Dox off d1 showed increased S_S_(AEinjure/alvBL); the injured fraction increased further in Dox off d3 (Figure 7B). This increase in S_S_(AEinjure/alvBL) occurred in concert with decreased S_S_(AE1/alvBL) while S_S_(AE2/alvBL) remained stable (Table 4). In order to avoid the reference trap, the surface fractions and S(alvBL,sep) were used to calculate absolute values of basal lamina covered by injured epithelial cells [S(AEinjure,sep)], AE1 cells S(AE1,sep) and AE2 cells S(AE2,sep). S(AEinjure,sep) and S(AE1,sep) were greater at higher Pao (Figure 7C,F). Moreover, S(AEinjure,sep) increased progressively from Dox on to Dox off d1 and Dox off d3 (Figure 7C). In order to describe the relationship between V(alvfluid,par) and S(AEinjure,sep) these data were plotted against each other. Since Pao influenced S(AEinjure,sep) this relationship was investigated for Pao = 2 cmH_2_O (Figure 7D). The distribution of points argued against a linear relationship. Hence, curve fitting was tested for an exponential or second order quadratic relationship between these two parameters using GraphPad PRISM statistic software (Version 7). The best curve fitting was achieved by an exponential growth equation:

(1) $$Y\left(x\right)\;=\;Y0\;\times \;e^{kx}$$

where Y(x) is the volume of alveolar fluid as a function of surface area of injured alveolar epithelium (x), Y0 is the volume of alveolar fluid with no injured alveolar surface, and *k* is the rate constant. In this equation best fit (*R^2^* = 0.77) was calculated for Y0 = 0.00028 cm^3^ and *k* = 0.044, suggesting an exponential relationship between V(alvfluid,par) and S(AEinjure,sep). Finally, S(AE2,sep) was not effected by Pao (Figure 7E). Although the factor group assignment was significantly influencing S(AE2,sep), the Tukey post-hoc adjustment of the p-level failed to reveal significant differences (Figure 7E).

### 2.5. Structure-function Relationships

In order to establish structure-function relationships, correlation analyses between structural and lung mechanical parameters were performed. The increase in H during PEEP = 2 cmH_2_O were correlated with S(AEinjure,sep) (*r* = 0.646, *p* = 0.002) but showed also correlations with parameters related to alveolar fluid such as V(alvfluid,par) (*r* = 0.548, *p* = 0.012), S(fluidAE,par) (*r* = 0.559, *p* = 0.01) and τ(alvfluid) (*r* = 0.54, *p* = 0.014). Cst (quasi-static compliance) and IC (inspiratory capacity) were characterized by an inverse correlation with S(AEinjure,sep) and the alveolar fluid related parameters. Hence, the more injured alveolar epithelial cells and the more alveolar fluid the less were Cst and IC. These relationships indicate mechanical stiffening and volume loss is associated with alveolar injury and fluid accumulation. In addition, V(alvair,par) was inversely correlated with H at PEEP = 2 cmH_2_O ventilation (*r* = −0.535, *p* = 0.015). During PEEP = 10 cmH_2_O ventilation the association of V(alvair,par) with lung mechanical parameters became stronger compared to PEEP = 2 cmH_2_O. For example, IC and V(alvair,par) were highly correlated with each other (r = 0.783, p < 0.001) while H and V(alvair,par) were inversely correlated (*r* = −0.516, *p* = 0.02). The central airway resistance Rn demonstrated a strong inverse correlation with V(alvair,par) (*r* = −0.596, *p* = 0.009) and S(airAE,sep) (*r* = −0.539, *p* = 0.014) and a positive correlation with S(AEinjure,sep) (*r* = 0.522, *p* = 0.018). These relationships indicate that the failure to recruit distal airspaces is linked to the abnormalities observed in the mechanical properties during PEEP = 10 cmH_2_O ventilation.

## 3. Discussion

The decrease in SP-B levels in BAL of patients represents an early event during the development of an ARDS [14] and correlates to the dysfunction of BAL-derived alveolar surfactant [16]. The contribution of this early decrease in BAL SP-B levels to lung injury is not entirely understood but it has been discussed that surfactant dysfunction represents a crucial step in the development of ARDS within a process known as ventilation induced lung injury (VILI) [17,38]. With this regard the repetitive opening of fluid occluded folds of alveolar walls in presence of high surface tension has been suggested to impose harmful forces on epithelial cells [22,39], a mechanism which can be referred to as microatelectrauma.

Mice expressing SP-B under the control of a doxycycline-dependent promotor demonstrated a decline in BAL SP-B levels within 24 h after withdrawal of doxycycline, a finding which correlated with a dramatic increase in minimum surface tension of BAL-derived surfactant [21]. In the present study we did not measure the surface function directly, e.g., by determining the minimum surface tension of BAL derived surfactant. At lung mechanical level, however, we observed an increase in hysteresis of the quasi-static PV-loop (Figure 1B) in Dox off d1 which occurred independently from signs of acute lung injury such as interstitial or alveolar edema formation. This finding can be interpreted as a result of increased surface tension. It has been well known for decades that the properties at the air-liquid interface are the main source of hysteresis related energy-loss during a PV-loop since the pressures to overcome surface tension during inspiration are increased with surface tension related elastic recoil pressure [4,7]. This finding is in line with the decrease in the inspiratory capacity (Figure 1C). Hence, the present study provides indirect evidence of high surface tension under quasi-static conditions during inspiration but we were not able to investigate whether or not high surface tension was present during dynamic breathing.

Further investigations in previous studies taking different time points after the induction of the knockout into account illustrated the occurrence of alveolar inflammation and increased BAL protein levels at day 3 [21] so that important criteria of acute lung injury were fulfilled in this animal model [9]. One goal of the present study was to investigate the time course of high surface tension related pathologies and their relationships, such as the link between alveolar micromechanics, alveolar fluid accumulation, and alveolar epithelial injury. In this context, the conditional SP-B knockout mouse model had a clear advantage compared to other models of direct or indirect lung injury [40]. Isolated high surface tension is the primary event and not a downstream consequence of an injurious trigger so this animal model allows investigation of the pure effect of high surface tension on disease initiation and progression. An important aspect of this study was that the recruitability of distal airspaces was investigated by examining morphometry at different airway pressures (Pao) using vascular perfusion fixation. With this approach, the effects of high surface tension on acinar microarchitecture could be investigated since the lung was air-filled at the time of fixation [35]. Although this is a static evaluation, the comparison of stereological parameters at different Pao provided information on the pressure-dependent dynamic changes in the recruitability of distal airspaces, an aspect of the so-called alveolar micromechanics, and the distribution and thickness of alveolar fluid [26].

### 3.1. Alveolar Micromechanics

One day after induction of SP-B deficiency discrete abnormalities in alveolar micromechanics were observed independent of an accumulation of alveolar fluid. These abnormalities did not significantly affect the organ-scale lung mechanical properties such as tissue elastance or quasi-static compliance but coincided with a modest decrease in the inspiratory capacity (Figure 1C). Abnormal alveolar micromechanics were characterized at a structural level by a significant reduction of volumes of alveolar airspaces at a Pao of 10 cmH_2_O in Dox off d1 compared to Dox on (Figure 3A), although, the number of open alveoli per lung did not differ between these two study groups (Figure 3D). Pressure-dependent alveolar volume changes can be attributed to septal wall stretching, changes in alveolar shape, septal wall folding and alveolar recruitment/derecruitment [5,35,41]. Since the surface area of the alveolar epithelial basal lamina (Table 4, Figure 7A), alveolar number and alveolar surface area (Figure 3) did not differ significantly between Dox on and Dox off d1 at Pao = 10 cmH_2_O there was no evidence of changes in alveolar septal wall stretching, folding, or alveolar derecruiment. Hence, it appeared to be likely that differences in alveolar shape were responsible for the high surface tension induced reduction in total volume of alveolar airspaces at Pao = 10 cmH_2_O.

Reducing the Pao from 10 to 2 cmH_2_O was linked with a substantial loss of alveolar surface area (*p* = 0.001) and total volume of alveolar airspace (*p* < 0.001) in all study groups while the number of open alveoli per lung was independent of Pao (Table 1, Figure 3D). In addition, the surface area of the alveolar epithelial basal lamina, a parameter used to quantify stretching of septal walls [6,42], was significantly affected by the factor Pao (Figure 7A, Table 4). Therefore, the alveolar-scale micromechanical mechanisms occurring with a drop in Pao from 10 to 2 cmH_2_O include folding of septal walls (Figure 2), alveolar shape changes and de-stretching of septal walls. Alveolar derecruitment seemed to play a minor role in volume changes and this was quite unexpected since the effect of surface tension on lung structure has been suggested to become most evident at low lung volumes, when surfactant function is critical for stabilization of distal airspaces [43]. Although surfactant dysfunction has been demonstrated to be present 24h after withdrawal of Doxycycline in this animal model [21] the loss of alveolar surface area at Pao = 2 cmH_2_O did not differ between Dox on and Dox off d1 in the present study. The absence of a relevant increase of alveolar instability in Dox off d1 was confirmed by the measurements of tissue elastance H which did not differ from data measured in Dox on during PEEP = 2 cmH_2_O ventilation (Figure 1D). An increase in H during low PEEP ventilation has been linked to alveolar derecruitment in previous studies [26,33,44,45].

Three days after induction of SP-B deficiency more severe abnormalities were observed compared to Dox on. A further reduction of the volume of alveolar airspaces was observed at both Pao = 2 cmH_2_O and 10 cmH_2_O. But the most striking observation in this group was that both the surface area of alveoli and the number of open alveoli were significantly reduced and this change was most pronounced at Pao = 10 cmH_2_O. Comparing Dox off d3 against Dox on and Dox off d1 it is evident that the increase in Pao from 2 to 10 cmH_2_O is associated with a failure to increase both the alveolar surface area and the number of open alveoli per lung (Figure 3C,D). This failure to recruit distal airspaces in Dox off d3 (Figure 2) occurs even though the minimum surface tension of BAL-derived, purified surfactant (so-called large aggregates) has been shown to remain constant in this time period [21]. Of note, the hysteresis of the quasi-static PV-loop in Dox off d3 did not differ from Dox on so that it can be speculated that the surface tension related increase in hysteresis in Dox off d1 is linked to recruitment of surface area, a process which is markedly impaired in a progressive state of lung injury at day 3 in this model.

### 3.2. Alveolar Fluid Properties and the Relationship to Alveolar Micromechanics

At ultrastructural level, the main difference between Dox off d3 and the other two groups was the dramatic increase in the volume of alveolar fluid per lung (Figure 5C). This increase was accompanied by higher BAL concentrations of protein and albumin (Figure 5A,B). The protein level in BAL fluid increased by the factor 2.2 (Figure 5A) while the stereological parameter, the absolute volume of alveolar fluid, increased by the factor 8.1 (Figure 5C). During vascular perfusion fixation of the lung for electron microscopy glutaraldehyde crosslinks the proteins which are located in the alveolar fluid so that the fluid becomes visible. In Dox off d3 the alveolar fluid was much darker in some areas of the lung compared to Dox on so that it can be concluded that the fluid was enriched with proteins. Taking the 8.1-fold increase in the volume of alveolar fluid into account the increase in BAL protein levels appears to be disproportionally low. While the stereological parameter is unbiased taking also the reference space into account, this is not the case regarding the protein level in BAL since the recovery of BAL fluid varied between 2 and 2.5 mL. Moreover, it can be speculated that during broncho-alveolar lavage the derecruited and edema filled distal airspaces could not be opened so that only recruitable parts were reached. This would result in a higher degree of dilution of alveolar proteins in Dox off d3 and therefore an underestimation of the increase in the amount of proteins within the alveolar space since for all lungs the same volume of fluid was instilled into the lungs.

Of note, the accumulation of alveolar fluid volume from 0.0009 cm^3^ in Dox on to 0.00734 cm^3^ in Dox off d3 was not clearly detectable at light microscopic level so that electron microscopic resolution was necessary for detailed quantitative assessments. In Dox off d3 the failure of recruitment of lung parenchyma could be observed at ultrastructural level in the surface area of alveolar epithelium covered by air (Figure 5E) or alveolar fluid (Figure 5F). In both Dox on and Dox off d1 a shift of surface areas from fluid covered to air covered alveolar epithelium was observed as Pao increased from 2 to 10 cmH_2_O. These pressure-dependent differences in alveolar airspace fluid distribution was coupled with an increase in the mean thickness of alveolar fluid (Figure 5D). At ultrastructural level, septal wall folds filled with a very thin leaflet of alveolar fluid were typical findings at low Pao while at higher Pao the fluid was concentrated in the corners of the alveoli. These observations suggest a pressure-dependent redistribution of fluid within the alveolar space, a mechanism which was linked with an unfolding of septal walls and therefore a recruitment of air-covered surface area. In Dox off d3, however, this pressure-dependent re-distribution of alveolar fluid was severely impaired as shown by the failure to shift the surface area of fluid-covered to air-covered alveolar epithelium with increase in Pao. In line with this observation was the fact that Pao had virtually no effect on the mean thickness of the alveolar fluid located on top of the alveolar epithelium in that group. Based on these observations it can be inferred that the surface tension and viscoelastic properties of the alveolar fluid differs substantially at day 3 of conditional SP-B knockout, leading to severe effects on alveolar micromechanics such as impaired alveolar recruitment. These alterations coincided with the increase in protein and albumin levels in BAL. It has been shown that distal airspace recruitability is impaired by increased alveolar protein [46] and fibrin levels [47], a finding which is in line with the structural data illustrated in the present study. In addition, the degradation of lung mechanical properties correlated with stereological data describing alveolar fluid and derecruitment of distal airspaces. This was also the case regarding the airway resistance (Rn) which usually decreases with PEEP due to outward tethering forces of elastic fibers that connect the conducting airways and the pleura [34,48]. In Dox off d3 the effect of increasing PEEP on airway resistance was less pronounced compared to the other groups (Figure 1E), an observation which can be explained by impaired recruitability of alveolar airspaces and therefore a reduction in the tethering forces on the conducting airways.

### 3.3. Injury of Alveolar Epithelium in the Context of impaired Alveolar Micromechanics and Fluid Accumulation

In both the Dox on and Dox off d1 groups the formation of septal wall pleats filled with a thin layer of fluid were observed with decreasing incidence with increasing Pao (Figure 4) so that it is possible that folding and unfolding of septal walls occurs during ventilation within the physiological range [5,41]. Computational simulations and in vitro experiments provided evidence that a finger-shaped bubble of air penetrating a fluid occluded airway is associated with harmful shear stresses resulting in necrosis of lining epithelial cells, an injurious event which can be prevented by addition of surfactant [22,49]. In the current study, an increase in the surface area of the basal lamina covered by injured epithelial cells was observed in Dox off d1 compared to Dox on (Figure 7C) and this was present even though the volume of alveolar fluid had not increased significantly (Figure 5C). Based on these observations we infer that the recruitment of airspaces by septal wall unfolding is an injurious event in Dox off d1 but not in Dox on, where the epithelium is protected from injury by functional surfactant. The injury of epithelial cells progresses in Dox off d3 to affecting approximately 6 to 8% of the surface area. A previous study using the LPS model of acute lung injury and light microscopic staining for AE1 cell markers found a loss of AE1 cells in 3% of the surface area [50]. In the present study, a complete denudation of the alveolar epithelial basal lamina was rarely observed and the main features related to injury were swelling and clearing of cytoplasmic ground substance, blebbing, and rupture of apical membranes (Figure 6). Among the structural parameters, the surface area of epithelial basal lamina covered by injured cells demonstrated a strong correlation with lung mechanical impairment, a finding which confirms observation in models of VILI where epithelial injury observed with scanning electron microscopy was strongly correlated with elastance [51]. Based on the presented data, the primary effects of induced SP-B deficiency are subtle alterations in alveolar micromechanics and alveolar epithelial injury that initially occur without abnormalities in the ultrastructural features of the alveolar fluid. The relationship between injury of the alveolar epithelial lining and the volume of alveolar fluid could best be described by an exponential growth function. The volume of alveolar fluid increased exponentially with the surface area of injured alveolar epithelium (Figure 7D). Similar relationships have been observed in a model of VILI between histologic injury scores and BAL protein levels [46] or after LPS injury during alveolar epithelial regeneration, plotting the percentage of regenerated alveolar epithelium against the BAL albumin level [50].

### 3.4. Limitations

In the present study assessment of lung structure was performed under “quasi-static” conditions at different airway pressures with the goal to characterize micromechanical aspects [26]. Also, fixed lung tissue was used for this purpose. While the microarchitecture was investigated by a robust methodology, the design-based stereology, up to the ultrastructural level, we did not study micromechanics directly. Instead, conclusions on the micromechanical behavior were made based on the comparison between parameters of microarchitecture at different airway pressures. Since the lung has viscoelastic properties the micromechanical behavior under dynamic breathing conditions is likely to differ from “quasi-static” conditions. For example nothing is known regarding the time scales at which folding and unfolding or fluid redistribution occurs. In vivo microscopy offers a powerful tool to study alveolar micromechanics under dynamic conditions [12,13]. However, it has to be pointed out that currently available imaging methods of in vivo microscopy are only able to study subpleural alveoli which might be not representative and do not have the appropriate resolution to investigate dynamics in fluid shift and unfolding of interalveolar septa since these aspects would require electron microscopic resolution [5]. In addition, female mice were used in the present study so that it cannot be excluded that a study using male mice would end up with different results and conclusion.

### 3.5. Summary

**Figure 8:**
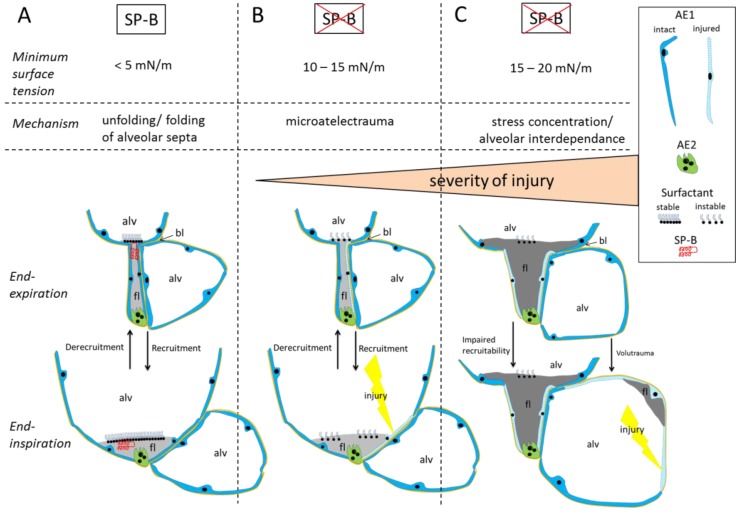
Proposed mechanism of lung injury in SP-B deficiency: Under physiological conditions (**A**) volume changes during ventilation include recruitment and derecruitment of folds. At low lung volumes folds are filled with a very thin leaflet of alveolar fluid (fl), the surfactant monolayer is compressed at the air-liquid interface and stabilized by SP-B. With increasing lung volume the folds are recruited and the fluid is pressed into the corners of the alveoli. The fluid-mechanical stress during this opening process acting on the alveolar epithelium is reduced by surface lowering properties of surfactant [22,39]. In absence of SP-B (indicated in B and C by the red cross) the monolayer is unstable, the minimum surface tension increases within 24 h [21]. The opening process of folds (**B**) is linked with harmful stresses acting on the alveolar epithelial cells (microatelectrauma) [22,39]. The blood-gas barrier is compromised by the progressing injury and the volume of alveolar fluid and plasma protein levels increase. High surface tension combined with increased viscosity of alveolar fluid reduces the recruitability of folds (**C**). Heterogeneous ventilation with overdistension of neighboring alveoli (alveolar interdependence) occurs with leakage of the blood-gas barrier in adjacent alveoli. Abbreviations: AE1: alveolar epithelial type 1 cell, AE2: alveolar epithelial type II cell, fl: alveolar fluid, bl: basal lamina, alv: alveolar airspace.

The presented data suggest that a kind of microatelectrauma might be the initial injurious event during spontaneous breathing in this animal model (Figure 8). We investigated the lung structure at low airway opening pressures and within a range of transpulmonary pressure gradients which is known to occur physiologically during quite breathing [52]. Hence, we consider the recruitment of pleats as a possible mechanism during spontaneous breathing. The unfolding of septal walls in the absence of SP-B might be responsible for alveolar epithelial cell injury in a manner similar to the reopening of fluid-occluded compliant airway models [22,36,49]. In the current study, the tissue strains resulting from peeling apart of septal pleats may be elevated by the same fluid-mechanical mechanisms that increase tissue strains during compliant airway reopening with high surface tension [39]. This injury of alveolar epithelium may occur prior to fluid accumulation and, in turn, increase alveolo-capillary permeability and lead to the observed exponential increase in alveolar fluid with increased alveolar protein and albumin levels. The combination of high surface tension and fluid accumulation results in a progressive alveolar derecruitment linked with a severely reduced recruitability of alveoli and increased ventilation heterogeneity. Alveolar interdependence with derecruited or edema filled alveoli next to ventilated alveoli has been shown to be a mechanism responsible for injurious overdistension of neighboring alveoli even at ventilation with low tidal volumes and pressures [30,53,54]. With this regard the forces acting on lung parenchyma are a consequence of the pressure gradient between the acinar airspaces and the pleural space, also known as elastic recoil pressure [25,55]. The elastic recoil pressure can be elevated during mechanical ventilation (high airway opening pressure), during spontaneous breathing (high negative pressure in pleural space) but also locally within a network of airspaces in the presence of stress concentrators. In principle, the latter can occur within a range of physiological pressure gradients at the organ scale and under spontaneous breathing. Hence, we consider non-recruitable distal airspaces as stress concentrators [25,56] which could end-up in a fatal vicious cycle following induction of SP-B deficiency. A similar scenario as we suggest for these spontaneously breathing SP-B deficient mice has been postulated as a rich-get-richer process in a model of alveolar leak during VILI. Accordingly, microatelectrauma represents the initial injurious trigger followed by a positive feedback mechanism of vascular leakage, edema formation, tethering-induced volutrauma, and further vascular leakage [46,57].

## 4. Materials and Methods

### 4.1. Animal Model and Study Groups

For pulmonary structural and mechanical investigations 40 female conditional Surfactant Protein B (SP-B) knock out mice [CCSP-rtTA, (tetO)_7_ *SFTPB/Sftpb*^-/-^] aged between 10 and 13 weeks were included. SP-B expression was under control of a doxycycline dependent promotor as has been described before [20,21]. The mice were randomly assigned to 3 groups. The control group (Dox on) was continuously fed with doxycycline containing food (625mg per kg standard diet, Altromin 1324, Lage, Germany). The experimental groups were deprived of doxycycline containing food for 1 and 3 days, respectively (Dox off d1 or Dox off d3) in order to investigate time effects of SP-B deficiency on lung structure and function. Animals assigned to Dox off d1 were transferred to cages with standard food on Mondays at 8:00 am. Lung mechanical properties were measured on Tuesdays between 8 and 10 am. The interval for Dox off d3 groups was from Monday 8:00 am to Thursday 8:00 am. In order to investigate the effects of airway opening pressures in these study groups, respiratory mechanics was evaluated during positive end-expiratory pressure (PEEP) ventilation with 2 and 10 cmH_2_O while lung structure was assessed at corresponding positive end-expiratory airway opening pressures (Pao). Hence, within each group animals were randomized to PEEP 2/Pao 2 cmH_2_O or PEEP 10/Pao 10 cmH_2_O. In addition, broncho-alveolar lavage (BAL) fluid was obtained from additional 17 animals (Dox on: *N* = 6 vs. Dox off d1: *N* = 6 vs. Dox off d3: *N* = 5). Since it is well-known that SP-B deficiency results in severe respiratory distress and death in this animal model starting at day 4 after withdrawal of Doxycycline containing food associated with a mortality rate of 40% [21], the number of subjects included in group Dox off d3 was reduced to the minimum, necessary to detect biologically relevant and statistically significant differences. The authorities of Lower Saxony, Germany (= LAVES: Niedersächsisches Landesamt für Verbraucherschutz und Lebensmittelsicherheit), which house the German equivalent of an institutional animal care and use committee, approved all animal experiments performed in this study according to the European Animal Welfare Regulations (Approval number: 16/2245).

### 4.2. Experimental Protocol

In order to investigate the respiratory mechanics during controlled ventilation in the different study groups (Dox on vs. Dox off 1d vs. Dox off 3d), mice were invasively ventilated by a FlexiVent rodent ventilator (SCIREQ, Montreal, PQ, Canada). The animals were anesthetized by intraperitoneal administration of 80 mg/kg bodyweight ketamine (Anesketin, Dechra Veterinary Products, Aulendorf, Germany), 5 mg/kg bodyweight xylazine (Rompun, Leverkusen, Germany) and 2mg/kg bodyweight midazolam. After disappearance of pain reflexes a tracheotomy was performed and the airways were connected to the FlexiVent rodent ventilator via a cannula (Braun cannula, 21 G, Diameter 0.8mm, Melsungen, Germany). Baseline ventilation parameters were as follows: tidal volume 10 mg/kg bodyweight, respiratory rate 150/min, inspiratory-to-expiratory time ratio 1:2 and PEEP 3 cmH_2_O. After a run-in phase of 5 min two deep inflations followed by three pressure-controlled pressure-volume (PV) loops were recorded to determine the inspiratory capacity, the quasi-static compliance of the respiratory system and the area within the PV-loop as a parameter of hysteresis [26]. Afterwards, PEEP was either adjusted to 2 cmH_2_O or 10 cmH_2_O followed by derecruitability tests as described elsewhere [19,26]. In brief, the derecruitability tests consisted of 2 recruitment manoeuvres (deep inflation with a pressure plateau at 30 cmH_2_O) and subsequent repetitive measurements of respiratory mechanics every 30 s using the forced oscillation technique. By fitting the constant phase model to impedance spectra obtained during forced oscillation technique, tissue elastance (H) and Newtonian resistance (Rn) were calculated. Tissue elastance is a parameter reflecting the mechanical properties of the fine lung parenchyma while Newtonian resistance is affected by alterations of the conducting airways and therefore the more central parts of the respiratory system. After recording tissue elastance for 5 min, the abdomen and chest wall was opened by a median laparo-thoracotomy. The lung was inflated to a plateau pressure of 30 cmH_2_O for 3 s two times (deep inflation) and airway opening pressure (Pao) was adjusted at either Pao of 2 or 10cmH_2_O on expiration, corresponding to the PEEP at which lung mechanics were assessed in each case [26].

### 4.3. Perfusion Fixation, Preparation and Sampling

The lungs were fixed by vascular perfusion at either Pao 2 or 10 cmH_2_O. The trachea was ligated at the corresponding Pao and flow of zero. The abdominal aorta was immediately incised and the right ventricle was punctured. The lungs were first flushed with a hydrostatic pressure of 40 cmH_2_O with Heparin (12,500 IU/l) in 0.9% sodium chloride solution to prevent coagulation in the pulmonary vasculature. Then, the fixative, consisting of 1.5% paraformaldehyde (PFA), 1.5% glutaraldehyde (GA) and 0.15 mM HEPES buffer, was perfused [45]. Finally, the organ package consisting of heart, lung, thymus and esophagus were carefully removed from the thoracic cavity and stored in the above mentioned fixation solution at 4 °C for at least 24 h.

For preparation and sampling, the lungs had to be carefully removed from the organ block using surgical scissors and tweezers. During the preparation, it was crucial to avoid compression of the lung to prevent contusing artifacts and artificial manipulation of the sensitive lung parenchyma. The heart, thymus, esophagus and fatty tissue were removed and the lungs were re-inserted in the fixation solution for at least 3 h.

The total lung volume [V(lung)] was determined based on the principle of Archimedes by the fluid displacement method as described by Scherle [58]. After measuring the lung volume, the whole lung was embedded in 4% agar and cut with a tissue slicer into slices of equal thickness from the apex to the base, enabling a “systematic uniform random sampling” [59]. The aim of “systematic uniform random sampling” was to give every part of the lung tissue the same chance of being selected for stereological investigation [60]. The tissue slicer generated 7 lung slices of an approximate thickness of 2 mm. Slices were randomly allocated to light (LM) or electron microscopy (EM).

Since the EM samples were limited in size, a subsampling of the slices assigned to EM was used to obtain 6-8 small cubes eligible to be further processed [59]. A point grid was thrown onto the lung slices. The lung tissue hit by the test points was selected and cut into cubes of 1 mm edge length with a scalpel knife and used for further EM embedding.

### 4.4. Embedding for Light Microscopy

The LM samples were embedded in hydroxyethylmethacrylate (Technovit 8100, Heraeus Kulzer, Wehrheim, Germany) in accordance to the user’s instructions. After washing in 0.2 mol Na-cacodylate buffer and osmification of the samples, the lungs were vented in a vacuum desiccator. After incubation in uranyl acetate overnight, dehydration was followed by an ascending acetone dilution series (70%, 90%, 100%). Then, the polymerization by the Technovit 8100 resulted in the curing of the samples. The rotation microtome (Leica, RM2265, Nussloch, Germany) was used for the slicing of the 1.5 µm thin sections. The first and the forth of a consecutive series of slices were placed on a glass slide, with Toluidine blue and capped with a cover glass [61].

### 4.5. Embedding for Electron Microscopy

The samples were washed in 0.15 HEPES buffer, post-fixed in osmium and contrasted *en-bloc* with uranyl acetate. Afterwards, dehydration was performed with an ascending acetone dilution series (70%, 90%, 100%). The probes were embedded in epoxy resin (Epon, Serva, Germany) in order to cut the samples into 60 nm thin slices employing the ultramicrotome (Leica, Nussloch, Germany),. Both the light microscopic and the electron microscopic embedding are based on the common procedures use in design-based stereological analyses [61].

### 4.6. Design-based Stereology

The methodology of stereology was applied to determine structural changes in the lungs as a function of the duration of SP-B deficiency (Dox on vs. Dox off d1 vs. Dox off d3) as well as the end-expiratory airway opening pressure (Pao = 2 cmH_2_O vs. Pao = 10 cmH_2_O). All methods for quantitative morphology used in the present study were based on the American Thoracic Society (ATS)/European Respiratory Society (ERS) joint statement for quantitative assessment of lung structures [62]. Design-based stereology provides information on 3-dimensional structures based on 2-dimensional sections which are combined with tests systems such as test points, test lines or counting frames interacting with the structures of interest in a stochastic manner [63]. The parameters determined at light and electron microscopic level are defined in Table 5.

The light microscopic assessments were carried out using a light microscope (Leica 6000, Wetzlar, Germany) equipped the computer-controlled stage and the NewCAST stereology software (Visiopharm A/S, Horshorm, Denmark) for systematic uniform area sampling and generation of an appropriate unbiased test-system for counting. The test system specified by the examiner was projected onto the sampled fields of view. The counting of events, defined as a stochastic interaction of the structures of interest with the test system was carried out by the blinded investigator. In order to ensure sufficient precision the unbiased test system was adjusted in a way that at least 100-200 counting events per parameter and organ were generated on 100 to 200 fields of view [64].

During the whole procedure, a cascade sampling design was applied [65]. In a first step, the lung was examined with a primary magnification of 5 × with the goal to distinguish between parenchymal and non-parenchymal components. Parenchyma was defined as fine lung structures which are directly involved in gas exchange while non-parenchyma was represented by conducting airways, larger vessels (excluding septal wall capillaries) and perivascular connective tissue. Test points were projected on fields of view. If a point hit structures involved in gas exchange (alveolar and ductal airspaces, interalveolar septa) it was counted as parenchyma [P(par)]. Points hitting bronchi, bronchiole, vessels or connective tissue were counted as non-parenchyma [P(nonpar)]. The following formula describes the volume density of parenchyma within the lung as a function of the total of all points hitting the lung (= reference space):

(2) $$Vv\;\left(\frac{par}{lung}\right)\;=\;\frac{\sum }{\sum P\left(par\right)\;+\;\sum P\;\left(nonpar\right)}$$

A final statement about the volume V of parenchymal and non-parenchymal components can only be taken if the result is related to the total lung volume. Otherwise there is a risk of falling into the “reference trap”, defined by Braendgaard and Gundersen [66]. Generally speaking, all generated densities must always be related to their reference volume in order to obtain absolute data, e.g., the total volume of lung parenchyma per lung. This avoids misinterpretations of densities or volume fractions due to differences in the reference space (= reference trap) [59].

(3) $$V\left(par,\;lung\right)\left(cm^{3}\right)\;=\;Vv\left(\frac{par}{lung}\right)\;\times \;V\left(lung\right)\left(cm^{3}\right)$$

In a second step of the cascade sampling design, the reference space was the lung parenchyma which was now assessed at higher magnification (primary magnification: 20 ×). The lung parenchyma was further differentiated into volumes of alveolar airspace V(alvair,lung), ductal airspaces V(ductair,lung) and interalveolar septa V(sep,lung) via point counting. The alveolar surface area S(alv,lung) was determined by counting the intersection points between a defined test line $\left(\frac{l}{p}\right)$ and the septa.

For example, the volume density of alveolar airspace within lung parenchyma was calculated using the following formula:

(4) $$Vv\left(\frac{alvair}{par}\right)\;=\;\frac{\sum P\left(alvair\right)}{\sum P\left(alvair\right)+\;\sum P\left(ductair\right)+\;\sum P\left(sep\right)}$$

The total volume of alveolar airspace was accordingly calculated by multiplication of the volume density with the reference space:

(5) $$V\left(alvair,lung\right)\left(cm^{3}\right)\;=\;Vv\left(\frac{alvair}{par}\right)\;\times \;V\left(par,\;lung\right)\left(cm^{3}\right)$$

The surface density of the alveoli within reference space (= lung parenchyma) was calculated as follows:

(6) $$Sv\left(\frac{alv}{par}\right)\left(cm^{-1}\right)\;=\;\frac{2\;\times \;\sum I}{\left(\frac{l}{p}\right)\left(\text{µ}m\right)\times \;P\left(line\right)}\;\times \;10.000$$

(7) $$\frac{l}{p}\left(\text{µ}m\right)\;=\;30.84$$

(8) P(line) = Points of the testline = reference points

The total surface area of alveoli at light microscopic level was accordingly determined by multiplication with the reference space:

(9) $$S\left(alv,\;lung\right)\left(cm^{2}\right)\;=\;Sv\left(\frac{alv}{Par}\right)(cm^{-1})\;\times \;V\left(par,lung\right)\left(cm^{3}\right)$$

In addition, the mean thickness of the septa was calculated from the ratio of the volume of the septa to the surface area.

(10) $$\tau \left(sep\right)\left(µm\right)\;=\;\frac{2\;\times \;V\left(sep,lung\right)\left(cm^{3}\right)\;\times \;10.000}{S\left(alv,lung\right)\left(cm^{2}\right)}$$

Furthermore, the number of open alveoli per lung N(alv,lung) [67,68] was calculated by employing the physical disector which consisted of a pair of section, corresponding to the first and the forth section of a consecutive series with a sections thickness of 1.5 µm. Hence, the distance from the top of the first to the top of the forth section was 4.5 µm representing the disector height. Using a counting frame with a defined area A(frame) a test volume for counting was generated. Whenever an alveolar mouth was present in one section and absent in the other, a counting event was recorded [67,69]. The estimate of the number of alveoli is based on the Euler number; a mathematical parameter (named after the swiss mathematician Leonard Euler), that quantifies the connectivity of an object. The Euler number [$x_{3}$] represents the number of alveolar openings (B).

(11) $$x_{3}=\;\sum B$$

(12) $$N\left(alv,lung\right)\;=\;\frac{\sum B\;}{disector\;height\;x\;A\left(frame\right)x\;\sum P\left(reference\;counting\;frame\right)}\times \;\;V\left(par,lung\right)$$

In the third step of the cascade sampling design, the volume of the interalveolar septa V(sep,lung) represented the reference space for further analyses at electron microscopic resolution. A transmission electron microscope (Morgani, FEI, Eindhoven, The Netherlands) equipped with an integrated camera (Olympus Soft Imaging Solution, Münster, Germany) was used to perform a systematic uniform area sampling at electron microscopic level. The section was traced meander-shaped via a coordinate system in x and y direction at a distance of 50 × 50 µm [59]. The septal wall was imaged every 50 µm using a primary magnification of 11,000x. Afterwards the sampled images were examined with the STEPanizer stereology tool [70]. The volume and surface densities were measured using unbiased test systems consisting of tests points for volume fractions of different structures and test lines for surface densities within the septal walls. The total volume of alveolar epithelial type I cells V(AE1,sep) and the total surface area of the alveolar epithelial basal lamina S(alvBL,sep) within the septal wall volume were for example calculated with the following equations:

(13) $$V\left(AE1,sep\right)\left(cm^{3}\right)\;=\;\frac{\sum P\left(AE1\right)}{\sum P\left(all\;counts\right)}\;\times \;V\left(sep,lung\right)\left(cm^{3}\right)$$

(14) $$S\left(alvBL,sep\right)\left(cm^{2}\right)\;=\;\frac{2\;x\;I\;x\;10.000}{l\left(t\right)x\;\sum P\left(all\;counts\right)}\left(cm^{-1}\right)\;\times \;V\left(sep,lung\right)\left(cm^{3}\right)$$

(15) test line = l(t)(µm) = 1.066 µm

(16) Intersection point between test line and interface of BL and AE = I

In addition, test lines were used to determine the surface area of the alveolar epithelial basal lamina (alvBL) covered by healthy AE1 or alveolar epithelial type 2 (AE2) cells or injured alveolar epithelial cells [19,71]. At the intersection of the test line with the alveolar epithelial basal lamina the topping epithelial cell was categorized as healthy appearing AE1 or AE2 cell or injured cell. The later was identified according the following criteria: swelling with formation of small vacuoles and clearing of the cytoplasmic ground substance, or fragmentation such as disruption of the apical plasma membrane, formation of vesicles up to denudation of the basal lamina. Data were calculated as surface fractions (in %) and absolute surface areas (in cm^2^).

### 4.7. Analyses of Broncho-alveolar Lavage Fluid

Animals were anaesthetized by intraperitoneal administration of 80 mg/kg bodyweight ketamine (Anesketin, Dechra Veterinary Products, Aulendorf, Germany), 5 mg/kg bodyweight xylazine (Rompun, Leverkusen, Germany) and 2 mg/kg bodyweight midazolam. After cessation of pain reflexes, the abdomen was opened and the abdominal aorta incised. Afterwards, a tracheotomy was carried out and the chest opened. For harvest of broncho-alveolar lavage (BAL) fluid three aliquots of 1 mL 0.9% sodium chloride solution were successively instilled and suctioned out of the lung. The range of recovery per lung was 2–2.5 mL. In order to separate cellular components and debris from fluid, a centrifugation with 1000g was carried out for 10 min. The supernatant was removed and snap frozen in liquid nitrogen and stored at –80 °C till further assessments of protein and albumin concentration as described previously [72]. In brief, albumin concentrations in BAL fluid were assessed with the albumin ELISA kit (Bethyl Laboratories, Montgomery, AL) according to the manufacturer’s instructions. Protein and albumin concentrations in BAL fluid served as indicators of vascular leak.

### 4.8. Statistical Analyses

A descriptive statistics was performed to calculate the mean and standard deviation of each parameter. A two-way ANOVA on ranks was carried out taking the factors “group” and the end-expiratory airway opening pressure (PEEP for lung mechanics and Pao for structural assessments) into account. In case of statistically significant differences, a Tukey´s post-hoc adjustment of the p-level for multiple testing was added. Regarding quasi-static compliance (Cst), hysteresis (area), inspiratory capacity (IC) and BAL data (protein and albumin) a one-way ANOVA was used followed by Tukey´s post-hoc test in case data were normally distributed according to Shapiro-Wilk normality test. Otherwise, a Kruskal-Wallis test followed by Dunn´s test for multiple comparisons was used. Pearson´s correlation analyses were performed between structural and lung mechanical parameters. V(alvfluid,par) and S(AEinjure,sep) were plotted against each other and the best fit curve was interpolated to describe the relationship between these parameters. Data are provided in the Tables as means and standard deviations while the graphs in the figures show dot blots of individual data for each group. Statistical assessments were performed using GraphPad Prism (Version 7.0, GraphPad Software, La Jolla, CA, USA) or in case of correlation analyses SPSS (Version 25, IBM, Armonk, NY, USA).

## Figures and Tables

**Table 1:** Lung architecture.

Group	Dox on		Dox off d1		Dox off d3		2wayANOVA	
Parameter	Pao 2	Pao 10	Pao 2	Pao 10	Pao 2	Pao 10	group	Pao
V(lung) cm^3^	0.52 (0.05)	1.18 (0.10)	0.42 (0.08)	0.95 (0.10)	0.40 (0.05)	0.80 (0.14)	< 0.01	< 0.01
V(alvair,lung) cm^3^	0.27 (0.02)	0.72 (0.07)	0.19 (0.05)	0.53 (0.08)	0.16 (0.03)	0.43 (0.08)	< 0.001	< 0.001
V(ductair,lung) cm^3^	0.05 (0.02)	0.19 (0.05)	0.04 (0.02)	0.16 (0.03)	0.04 (0.01)	0.14 (0.02)	n.s.	< 0.01
V(sep,lung) cm^3^	0.13 (0.02)	0.12 (0.02)	0.12 (0.01)	0.13 (0.03)	0.13 (0.01)	0.12 (0.02)	n.s.	n.s.
S(alv,lung) cm^2^	268 (83.0)	374 (110)	272 (55.0)	395 (50.0)	205 (26.7)	254 (72.6)	0.008	0.001
τ (sep) µm	10.3 (2.91)	6.77 (1.04)	9.37 (1.24)	6.53 (1.70)	13.2 (1.19)	10.2 (2.91)	< 0.001	< 0.001
N(alv,lung) 10^6^	1.75 (0.81)	2.61 (1.01)	2.31 (0.63)	2.80 (0.39)	1.47 (0.21)	1.47 (0.52)	0.005	n.s.

Data are given as mean (SD). n.s.: not significant. Parameters are detailed in the text and Table 5.

**Table 2:** Composition of interalveolar septa.

	Dox on		Dox off d1		Dox off d3		2wayANOVA	
Parameter	Pao 2	Pao 10	Pao 2	Pao 10	Pao 2	Pao 10	Group	Pao
V(AE1,sep) mm^3^	15.2 (2.01)	15.4 (3.19)	11.6 (2.00)	16.5 (3.14)	14.4 (0.97)	13.0 (1.86)	n.s.	n.s.
V(AE2,sep) mm^3^	4.92 (1.36)	7.73 (3.01)	6.24 (1.66)	6.78 (2.20)	5.96 (2.58)	4.67 (2.00)	n.s.	n.s.
V(endo,sep) mm^3^	20.8 (1.58)	20.7 (2.94)	18.7 3.42	21.3 (4.97)	20.2 (1.68)	20.1 (2.89)	n.s.	n.s.
V(othercells,sep) mm^3^	8.04 (1.95)	9.02 (2.07)	8.01 (1.38)	12.6 (5.49)	9.05 (0.8)	9.12 (1.4)	n.s.	n.s.
V(ECM,sep) mm^3^	3.78 (0.95)	4.15 (1.65)	3.60 (0.73)	6.42 (2.99)	3.52 (0.79)	4.69 (2.30)	n.s.	0.024
V(caplumen,sep) mm^3^	73.7 (16.3)	63.7 (15.2)	75.3 (8.89)	57.5 (12.5)	73.04 (9.34)	63.05 (10.8)	n.s.	0.009
S(endoBL,sep) cm^2^	775 (138)	915 (188)	700 (81.0)	804 (175)	689 (76.3)	739 (83.5)	n.s.	0.049

Data are given as mean (SD). n.s.: not significant. Parameters are defined in the text and in Table 5.

**Table 3:** Alveolar fluid.

	Dox on		Dox off d1		Dox off d3		2wayANOVA	
Parameter	Pao 2	Pao 10	Pao 2	Pao 10	Pao 2	Pao 10	Group	Pao
V(alvfluid,par) mm^3^	0.79 (0.32)	1.39 (0.95)	0.92 (0.26)	6.64 (11.4)	8.03 (3.95)	6.63 (3.95)	0.032	n.s.
S(airAE,par) cm^2^	401 (103)	717 (112)	320 (76.2)	628 (190)	200 (60.0)	310 (89.2)	< 0.001	< 0.001
S(fluidAE,par) cm^2^	353 (58.7)	236 (55.3)	366 (29.4)	280 (113)	483 (39.3)	393 (101)	< 0.001	< 0.001
τ(alvfluid) nm	39.0 (18.6)	89.0 (48.0)	44.2 (16.6)	248 (294)	297 (155)	268 (125)	0.006	n.s.

Data are given as mean (SD). n.s.: not significant. Parameters are defined in Table 5.

**Table 4:** Injury of alveolar epithelium.

	Dox on		Dox off d1		Dox off d3		2wayANOVA	
Parameter	Pao 2	Pao 10	Pao 2	Pao 10	Pao 2	Pao 10	Group	Pao
S(alvBL,sep) cm^2^	775 (129)	954 (181)	686 (82.4)	879 (206)	660 (76.3)	727 (105)	0.034	0.006
S_S_(AEinjure/alvBL)%	0.97 (0.76)	0.86 (0.55)	2.78 (0.99)	4.03 (1.39)	6.83 (2.54)	8.94 (4.12)	< 0.001	n.s.
S_S_(AE1/alvBL)%	93.3 (1.27)	94.0 (2.15)	90.1 (2.10)	89.4 (2.30)	88.0 (3.44)	86.4 (3.45)	< 0.001	n.s.
S_S_(AE2/alvBL)%	5.16 (1.31)	5.09 (2.37)	7.15 (2.21)	6.59 (1.70)	5.21 (1.18)	4.67 (1.73)	0.033	n.s.
S(AEinjure,sep) cm^2^	7.31 (5.48)	7.87 (5.13)	19.0 (4.07)	34.4 (14.1)	44.1 (14.4)	67.7 (37.6)	< 0.001	0.025
S(AE1,sep) cm^2^	728 (126)	900 (184)	617 (62.6)	786 (188)	582 (84.9)	626 (78.2)	0.004	0.011
S(AE2,sep) cm^2^	39.5 (9.99)	46.4 (22.7)	50.1 (20.1)	58.4 (20.1)	33.7 (4.97)	33.5 (12.0)	0.03	n.s.

Data are given as mean (SD). n.s.: not significant. S_S_: surface fraction. Parameters are defined in the text and in Table 5.

**Table 5:** Definition of stereological parameters.

Definition	Abbreviation	Test System	Magnification
Volume of parenchyma	V(par,lung)	Point counting (P)	5 ×
Volume of non-parenchyma (conducting airways, larger vessels, connective tissue)	V(nonpar,lung)	Point counting (P)	5 ×
Volume of alveolar airspaces within lung parenchyma	V(alvair,lung)	Point counting(P)	20 ×
Volume of ductal airspaces within lung parenchyma	V(ductair,lung)	Point counting (P)	20 ×
Volume of interalveolar septa within lung parenchyma	V (sep,lung)	Point counting (P)	20 ×
Surface area of alveoli within lung parenchyma	S (alv,lung)	Intersection counting (I)	20 ×
Arithmetic mean septal wall thickness	τ(sep)	Volume-to-surface ratio	20 ×
Number alveoli within lung parenchyma	N(alv,lung)	Physical disector	10 ×
Volume of alveolar epithelial type 1 cells within septal walls	V(AE1,sep)	Point counting (P)	11.000 ×
Volume of alveolar epithelial type 2 cells within septal walls	V(AE2,sep)	Point counting (P)	11.000 ×
Volume of endothelial cell within septal walls	V(endo,sep)	Point counting (P)	11.000 ×
Volume of other cells (e.g., macrophages, fibroblasts)	V(othercells,sep)	Point counting (P)	11.000 ×
Volume of extracellular matrix within septal walls	V(ECM,sep)	Point counting (P)	11.000 ×
Volume of capillary lumen within septal walls	V(cap,sep)	Point counting (P)	11.000 ×
Volume of alveolar fluid within parenchyma	V(alvfluid,par)	Point counting (P)	11.000 ×
Surface area of the alveolar epithelial basal lamina within septal walls	S(alvBL,sep)	Intersection counting (I)	11.000 ×
Surface area of the basal lamina covered by AE1 cells	S(AE1,sep)	Intersection counting (I)	11.000 ×
Surface area of the basal lamina covered by AE2 cells	S(AE2,sep)	Intersection counting (I)	11.000 ×
Surface area of the basal lamina covered by injured alveolar epithelial cells	S(AEinjure,sep)	Intersection counting (I)	11.000 ×
Surface area of the endothelial basal lamina within septal walls	S(endoBL,sep)	Intersection (I) counting	11.000 ×
Surface area of the alveolar epithelium covered by air within parenchyma	S(airAE,par)	Intersection (I) counting	11.000 ×
Surface area of alveolar epithelium covered by alveolar fluid within parenchyma	S(fluidAE,par)	Intersection (I) counting	11.000 ×
Arithmetic mean thickness of alveolar fluid	τ(alvfluid)	Volume-to-surface ratio	11.000 ×

## References

[1] Lopez-Rodriguez E., Gay-Jordi G., Mucci A., Lachmann N., Serrano-Mollar A. (2017). Lung surfactant metabolism: Early in life, early in disease and target in cell therapy. Cell Tissue Res..

[2] Schürch D., Ospina O.L., Cruz A., Pérez-Gil J. (2010). Combined and independent action of proteins SP-B and SP-C in the surface behavior and mechanical stability of pulmonary surfactant films. Biophys. J..

[3] Ochs M. (2010). The closer we look the more we see? Quantitative microscopic analysis of the pulmonary surfactant system. Cell Physiol. Biochem..

[4] Mead J. (1961). Mechanical properties of lungs. Physiol. Rev..

[5] Knudsen L., Ochs M. (2018). The micromechanics of lung alveoli: Structure and function of surfactant and tissue components. Histochem. Cell Biol..

[6] Bachofen H., Schürch S., Urbinelli M., Weibel E. (1987). Relations among alveolar surface tension, surface area, volume, and recoil pressure. J. Appl. Physiol. (1985).

[7] Schürch S., Bachofen H., Possmayer F. (2001). Surface activity in situ, in vivo, and in the captive bubble surfactometer. Comp. Biochem. Physiol. A Mol. Integr. Physiol..

[8] Almlén A., Stichtenoth G., Linderholm B., Haegerstrand-Björkman M., Robertson B., Johansson J., Curstedt T. (2008). Surfactant proteins B and C are both necessary for alveolar stability at end expiration in premature rabbits with respiratory distress syndrome. J. Appl. Physiol. (1985).

[9] Matute-Bello G., Downey G., Moore B.B., Groshong S.D., Matthay M.A., Slutsky A.S., Kuebler W.M., Group A.L.I.i.A.S. (2011). An official American Thoracic Society workshop report: Features and measurements of experimental acute lung injury in animals. Am. J. Respir. Cell Mol. Biol..

[10] Cabrera-Benítez N.E., Parotto M., Post M., Han B., Spieth P.M., Cheng W.E., Valladares F., Villar J., Liu M., Sato M. (2012). Mechanical stress induces lung fibrosis by epithelial-mesenchymal transition. Crit. Care Med..

[11] Wilson T.A., Bachofen H. (1982). A model for mechanical structure of the alveolar duct. J. Appl. Physiol. (1985).

[12] Schiller H.J., McCann U.G., Carney D.E., Gatto L.A., Steinberg J.M., Nieman G.F. (2001). Altered alveolar mechanics in the acutely injured lung. Crit. Care Med..

[13] Tabuchi A., Nickles H.T., Kim M., Semple J.W., Koch E., Brochard L., Slutsky A.S., Pries A.R., Kuebler W.M. (2016). Acute Lung Injury Causes Asynchronous Alveolar Ventilation That Can Be Corrected by Individual Sighs. Am J. Respir. Crit. Care Med..

[14] Greene K., Wright J., Steinberg K., Ruzinski J., Caldwell E., Wong W., Hull W., Whitsett J., Akino T., Kuroki Y. (1999). Serial changes in surfactant-associated proteins in lung and serum before and after onset of ARDS. Am. J. Respir Crit. Care Med..

[15] Gregory T.J., Longmore W.J., Moxley M.A., Whitsett J.A., Reed C.R., Fowler A.A., Hudson L.D., Maunder R.J., Crim C., Hyers T.M. (1991). Surfactant chemical composition and biophysical activity in acute respiratory distress syndrome. J. Clin. Investig..

[16] Schmidt R., Markart P., Ruppert C., Wygrecka M., Kuchenbuch T., Walmrath D., Seeger W., Guenther A. (2007). Time-dependent changes in pulmonary surfactant function and composition in acute respiratory distress syndrome due to pneumonia or aspiration. Respir. Res..

[17] Albert R.K. (2012). The role of ventilation-induced surfactant dysfunction and atelectasis in causing acute respiratory distress syndrome. Am. J. Respir. Crit. Care Med..

[18] Nieman G.F., Bredenberg C.E. (1985). High surface tension pulmonary edema induced by detergent aerosol. J. Appl. Physiol..

[19] Lutz D., Gazdhar A., Lopez-Rodriguez E., Ruppert C., Mahavadi P., Gunther A., Klepetko W., Bates J.H., Smith B., Geiser T. (2015). Alveolar Derecruitment and Collapse Induration as Crucial Mechanisms in Lung Injury and Fibrosis. Am. J. Respir. Cell Mol. Biol..

[20] Nesslein L.L., Melton K.R., Ikegami M., Na C.L., Wert S.E., Rice W.R., Whitsett J.A., Weaver T.E. (2005). Partial SP-B deficiency perturbs lung function and causes air space abnormalities. Am. J. Physiol. Lung Cell Mol. Physiol..

[21] Ikegami M., Whitsett J.A., Martis P.C., Weaver T.E. (2005). Reversibility of lung inflammation caused by SP-B deficiency. Am. J. Physiol. Lung Cell Mol. Physiol..

[22] Bilek A.M., Dee K.C., Gaver D.P. (2003). Mechanisms of surface-tension-induced epithelial cell damage in a model of pulmonary airway reopening. J. Appl. Physiol. (1985).

[23] Hobi N., Ravasio A., Haller T. (2012). Interfacial stress affects rat alveolar type II cell signaling and gene expression. Am J. Physiol. Lung Cell Mol. Physiol..

[24] Ravasio A., Hobi N., Bertocchi C., Jesacher A., Dietl P., Haller T. (2011). Interfacial sensing by alveolar type II cells: A new concept in lung physiology?. Am. J. Physiol. Cell Physiol..

[25] Mead J., Takishima T., Leith D. (1970). Stress distribution in lungs: A model of pulmonary elasticity. J. Appl. Physiol..

[26] Knudsen L., Lopez-Rodriguez E., Berndt L., Steffen L., Ruppert C., Bates J.H.T., Ochs M., Smith B.J. (2018). Alveolar Micromechanics in Bleomycin-induced Lung Injury. Am. J. Respir. Cell Mol. Biol..

[27] Slutsky A.S., Ranieri V.M. (2013). Ventilator-induced lung injury. N. Engl. J. Med..

[28] Glindmeyer H.W., Smith B.J., Gaver D.P. (2012). In situ enhancement of pulmonary surfactant function using temporary flow reversal. J. Appl. Physiol..

[29] Ramsingh R., Grygorczyk A., Solecki A., Cherkaoui L.S., Berthiaume Y., Grygorczyk R. (2011). Cell deformation at the air-liquid interface induces Ca^2+^-dependent ATP release from lung epithelial cells. Am. J. Physiol. Lung Cell Mol. Physiol..

[30] Wu Y., Kharge A.B., Perlman C.E. (2014). Lung ventilation injures areas with discrete alveolar flooding, in a surface tension-dependent fashion. J. Appl. Physiol. (1985).

[31] Hantos Z., Daroczy B., Suki B., Nagy S., Fredberg J.J. (1992). Input impedance and peripheral inhomogeneity of dog lungs. J. Appl. Physiol..

[32] Allen G.B., Pavone L.A., DiRocco J.D., Bates J.H., Nieman G.F. (2005). Pulmonary impedance and alveolar instability during injurious ventilation in rats. J. Appl. Physiol. (1985).

[33] Massa C.B., Allen G.B., Bates J.H. (2008). Modeling the dynamics of recruitment and derecruitment in mice with acute lung injury. J. Appl. Physiol..

[34] Paré P.D., Mitzner W. (2012). Airway-parenchymal interdependence. Compr. Physiol..

[35] Gil J., Bachofen H., Gehr P., Weibel E. (1979). Alveolar volume-surface area relation in air- and saline-filled lungs fixed by vascular perfusion. J. Appl. Physiol. (1985).

[36] Gaver D.P., Halpern D., Jensen O.E., Grotberg J.B. (1996). The steady motion of a semi-infinite bubble through a flexible-walled channel. J. Fluid Mech..

[37] Kay S.S., Bilek A.M., Dee K.C., Gaver D.P. (2004). Pressure gradient, not exposure duration, determines the extent of epithelial cell damage in a model of pulmonary airway reopening. J. Appl. Physiol..

[38] Nieman G.F., Gatto L.A., Habashi N.M. (2015). Impact of mechanical ventilation on the pathophysiology of progressive acute lung injury. J. Appl. Physiol. (1985).

[39] Naire S., Jensen O.E. (2005). Epithelial cell deformation during surfactant-mediated airway reopening: A theoretical model. J. Appl. Physiol. (1985).

[40] Matute-Bello G., Frevert C.W., Martin T.R. (2008). Animal models of acute lung injury. Am. J. Physiol. Lung Cell Mol. Physiol..

[41] Roan E., Waters C.M. (2011). What do we know about mechanical strain in lung alveoli?. Am. J. Physiol. Lung Cell Mol. Physiol..

[42] Tschumperlin D.J., Margulies S.S. (1999). Alveolar epithelial surface area-volume relationship in isolated rat lungs. J. Appl. Physiol. (1985).

[43] Bachofen H., Schürch S. (2001). Alveolar surface forces and lung architecture. Comp. Biochem. Physiol. A Mol. Integr. Physiol..

[44] Smith B.J., Grant K.A., Bates J.H. (2013). Linking the development of ventilator-induced injury to mechanical function in the lung. Ann. Biomed. Eng..

[45] Beike L., Wrede C., Hegermann J., Lopez-Rodriguez E., Kloth C., Gauldie J., Kolb M., Maus U.A., Ochs M., Knudsen L. (2019). Surfactant dysfunction and alveolar collapse are linked with fibrotic septal wall remodeling in the TGF-β1-induced mouse model of pulmonary fibrosis. Lab. Investig..

[46] Smith B.J., Bartolak-Suki E., Suki B., Roy G.S., Hamlington K.L., Charlebois C.M., Bates J.H.T. (2017). Linking Ventilator Injury-Induced Leak across the Blood-Gas Barrier to Derangements in Murine Lung Function. Front. Physiol..

[47] Allen G.B., Leclair T., Cloutier M., Thompson-Figueroa J., Bates J.H. (2007). The response to recruitment worsens with progression of lung injury and fibrin accumulation in a mouse model of acid aspiration. Am. J. Physiol. Lung Cell Mol. Physiol..

[48] Weibel E. (2009). What makes a good lung?. Swiss Med. Wkly..

[49] Ghadiali S., Huang Y. (2011). Role of airway recruitment and derecruitment in lung injury. Crit. Rev. Biomed. Eng..

[50] Jansing N.L., McClendon J., Henson P.M., Tuder R.M., Hyde D.M., Zemans R.L. (2017). Unbiased Quantitation of Alveolar Type II to Alveolar Type I Cell Transdifferentiation during Repair after Lung Injury in Mice. Am. J. Respir. Cell Mol. Biol..

[51] Hamlington K.L., Smith B.J., Dunn C.M., Charlebois C.M., Roy G.S., Bates J.H.T. (2018). Linking lung function to structural damage of alveolar epithelium in ventilator-induced lung injury. Respir. Physiol. Neurobiol..

[52] Fredberg J.J., Kamm R.D. (2006). Stress transmission in the lung: Pathways from organ to molecule. Annu. Rev. Physiol..

[53] Perlman C.E., Lederer D.J., Bhattacharya J. (2011). Micromechanics of alveolar edema. Am. J. Respir. Cell Mol. Biol..

[54] Schirrmann K., Mertens M., Kertzscher U., Kuebler W.M., Affeld K. (2010). Theoretical modeling of the interaction between alveoli during inflation and deflation in normal and diseased lungs. J. Biomech..

[55] Loring S.H., Topulos G.P., Hubmayr R.D. (2016). Transpulmonary Pressure: The Importance of Precise Definitions and Limiting Assumptions. Am. J. Respir. Crit. Care Med..

[56] Albert R.K., Smith B., Perlman C.E., Schwartz D.A. (2019). Is Progression of Pulmonary Fibrosis due to Ventilation-induced Lung Injury?. Am. J. Respir. Crit. Care Med..

[57] Hamlington K.L., Bates J.H.T., Roy G.S., Julianelle A.J., Charlebois C., Suki B., Smith B.J. (2018). Alveolar leak develops by a rich-get-richer process in ventilator-induced lung injury. PLoS ONE.

[58] Scherle W. (1970). A simple method for volumetry of organs in quantitative stereology. Mikroskopie.

[59] Tschanz S., Schneider J.P., Knudsen L. (2014). Design-based stereology: Planning, volumetry and sampling are crucial steps for a successful study. Ann. Anat..

[60] Ochs M., Mühlfeld C. (2013). Quantitative microscopy of the lung: A problem-based approach. Part 1: Basic principles of lung stereology. Am. J. Physiol. Lung Cell Mol. Physiol..

[61] Muhlfeld C., Knudsen L., Ochs M. (2013). Stereology and morphometry of lung tissue. Methods in Mol. Biol. (Clifton, N.J.).

[62] Hsia C., Hyde D., Ochs M., Weibel E. (2010). An official research policy statement of the American Thoracic Society/European Respiratory Society: Standards for quantitative assessment of lung structure. Am. J. Respir. Crit. Care Med..

[63] Weibel E., Hsia C., Ochs M. (2007). How much is there really? Why stereology is essential in lung morphometry. J. Appl. Physiol..

[64] Gundersen H., Jensen E. (1987). The efficiency of systematic sampling in stereology and its prediction. J. Microsc..

[65] Ochs M. (2006). A brief update on lung stereology. J. Microsc..

[66] Braendgaard H., Gundersen H.J. (1986). The impact of recent stereological advances on quantitative studies of the nervous system. J. Neurosci. Methods.

[67] Ochs M., Nyengaard L.R., Lung A., Knudsen L., Voigt M., Wahlers T., Richter J., Gundersen H.J.G. (2004). The number of alveoli in the human lung. Am. J. Respir. Crit. Care Med..

[68] Gundersen H., Bagger P., Bendtsen T., Evans S., Korbo L., Marcussen N., Møller A., Nielsen K., Nyengaard J., Pakkenberg B. (1988). The new stereological tools: Disector, fractionator, nucleator and point sampled intercepts and their use in pathological research and diagnosis. APMIS.

[69] Hyde D., Tyler N., Putney L., Singh P., Gundersen H. (2004). Total number and mean size of alveoli in mammalian lung estimated using fractionator sampling and unbiased estimates of the Euler characteristic of alveolar openings. Anat. Rec. A Discov. Mol. Cell Evol. Biol..

[70] Tschanz S.A., Burri P.H., Weibel E.R. (2011). A simple tool for stereological assessment of digital images: The STEPanizer. J. Microsc..

[71] Fehrenbach H., Schepelmann D., Albes J., Bando T., Fischer F., Fehrenbach A., Stolte N., Wahlers T., Richter J. (1999). Pulmonary ischemia/reperfusion injury: A quantitative study of structure and function in isolated heart-lungs of the rat. Anat. Rec..

[72] Steffen L., Ruppert C., Hoymann H.G., Funke M., Ebener S., Kloth C., Mühlfeld C., Ochs M., Knudsen L., Lopez-Rodriguez E. (2017). Surfactant replacement therapy reduces acute lung injury and collapse induration-related lung remodeling in the bleomycin model. Am. J. Physiol. Lung Cell Mol. Physiol..

